# Is the Juice Worth the Squeeze? Vitamin C Supplementation in Hemodialysis Patients: A Systematic Review

**DOI:** 10.3390/nu18050774

**Published:** 2026-02-27

**Authors:** Małgorzata Sikorska-Wiśniewska, Magdalena Jankowska, Leszek Tylicki, Alicja Dębska-Ślizień

**Affiliations:** Department of Nephrology, Transplantology and Internal Medicine, Faculty of Medicine, Medical University of Gdansk, 80-210 Gdansk, Poland; magdalena.jankowska@gumed.edu.pl (M.J.); adeb@gumed.edu.pl (A.D.-Ś.)

**Keywords:** vitamin C, ascorbic acid, hemodialysis, end-stage renal disease

## Abstract

**Background:** Patients undergoing hemodialysis commonly exhibit deficiencies in water-soluble vitamins, primarily as a result of inadequate dietary intake and loss into the dialysate. Given the essential role of vitamin C in numerous metabolic pathways, routine supplementation has been proposed as a potentially beneficial intervention in this population. **Aim:** We aimed to evaluate the current evidence on vitamin C supplementation in patients undergoing hemodialysis, with particular attention to clinical conditions associated with renal replacement therapy, including anemia, chronic inflammation, restless legs syndrome (RLS), and secondary hyperparathyroidism. **Methods:** This systematic review was conducted in accordance with PRISMA guidelines. The MEDLINE (via PubMed) and EMBASE databases were searched. The initial search yielded 844 articles, of which 37 studies met the inclusion criteria for this review. **Results:** Evidence indicates that hemodialysis patients exhibit vitamin C deficiency, both in dietary intake and in plasma or serum concentrations. Despite its intrinsic antioxidant properties and proposed anti-inflammatory effects, vitamin C supplementation has demonstrated inconsistent effects on inflammatory markers. Most clinical studies support a beneficial role of vitamin C supplementation in functional iron deficiency and in alleviating symptoms of RLS within this population. **Conclusions**: Evidence on vitamin C supplementation for functional iron deficiency and RLS suggests that it might be an effective therapeutic approach. However, despite low serum vitamin C level in hemodialysis patients, current data does not justify the routine use of vitamin C in the hemodialyzed population for other comorbidities, including chronic inflammation and secondary hyperparathyroidism. Further high-quality studies are required to establish the broader clinical utility of targeted vitamin C supplementation.

## 1. Introduction

Patients with advanced kidney disease undergoing hemodialysis (HD) experience numerous complications related to renal replacement therapy, including vitamin deficiency. These patients are at particularly high risk of water-soluble vitamin deficiency, including vitamin C, largely due to their loss into the dialysate. Low levels of ascorbic acid (vitamin C) has been linked to comorbidities such as anemia, chronic inflammation, secondary hyperparathyroidism, restless legs syndrome (RLS), and cardiovascular disease, potentially worsening their clinical course. Given its central role as an antioxidant, vitamin C deficiency can exacerbate reactive oxygen species (ROS)-mediated oxidative stress, leading to oxidative modifications of proteins that alter their structure, immunogenicity, and function, thereby contributing to disease onset, progression, and immune dysregulation [1]. HD patients are considered to have inadequate dietary intake of vitamin C and are reported to exhibit lower intake than those on peritoneal dialysis or after kidney transplantation [2]. Historically, the clinical consequences of vitamin C deficiency were first described in 1974, when a Scottish physician reported cases of scurvy among sailors deprived of citrus fruit. Interestingly, freshly squeezed orange juice contains approximately 125 mg of vitamin C, whereas store-bought juice contains approximately 40–60 mg [3]. Currently, there is no consensus on vitamin C supplementation in chronic kidney disease (CKD) patients. It is worth noticing that the dialysis population is characterized by a significantly higher dietary supplements use in comparison to other CKD stages [4]. Kidney Disease Outcomes Quality Initiative (KDOQI) guidelines from 2020 suggest a daily vitamin C intake of 90 mg/d for men and 75 mg/d for women in patients with CKD [5]. KDIGO guidelines from 2012 do not recommend vitamin C supplementation due to an insufficient number of studied patients to address the safety [6].

There is considerable clinical uncertainty regarding the role of vitamin C in patients undergoing HD. A systematic review of studies evaluating vitamin C supplementation was conducted to assess the current state of knowledge in this field. We aimed to investigate reports on dietary intake adequacy, plasma or serum vitamin C concentrations, and their effects on oxidative stress, inflammation, anemia management, RLS, and other clinical outcomes in hemodialyzed patients. In this systematic review, we summarize actual knowledge and studies performed in the last 15 years.

## 2. Materials and Methods

**Figure 1:**
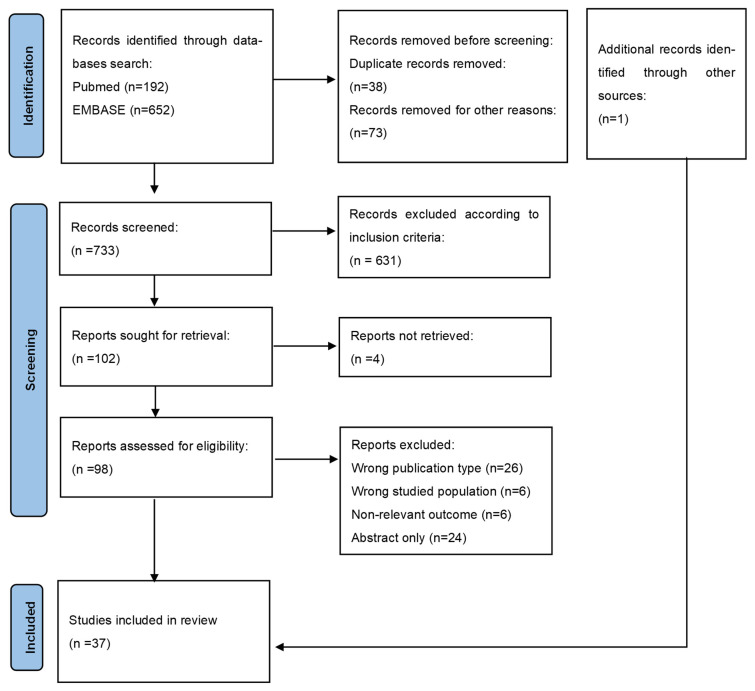
From: Page, M.J.; McKenzie, J.E.; Bossuyt, P.M.; Boutron, I.; Hoffmann, T.C.; Mulrow, C.D.; Shamseer, L.; Tetzlaff, J.M.; Akl, E.A.; Brennan, S.E.; et al. *BMJ* **2021**, *372*, n71. doi: 10.1136/bmj.n71 [7].

The present review was conducted in accordance with the PRISMA (Preferred Reporting Items for Systematic Reviews and Meta-Analyses) guidelines [7]. A comprehensive literature search was performed on 10 November 2025 using the Embase and MEDLINE via PubMed database. Two independent researchers (M.S.W. and M.J.) retrieved and summarized information from the eligible studies in tables. The following MeSH terms and keywords were applied: hemodialysis AND ascorbic acid OR vitamin C. The search was limited to studies published in English between 2010 and 2025. Inclusion criteria were human studies, adult population and intermittent form of hemodialysis. Both intravenous and oral vitamin C supplementation studies were eligible for inclusion. Studies involving patients receiving conservative management of chronic kidney disease, peritoneal dialysis, or continuous forms of renal replacement therapy were excluded. Studies in which patients treated with different renal replacement therapy modalities were pooled into a single study group were also excluded. Abstracts, case reports and studies with substantial methodological limitations were also excluded. Due to substantial heterogeneity in study designs and outcome measures, a formal risk-of bias assessment was not conducted. Instead, study characteristics and outcomes were transparently reported in tables to enable quality comparison. A detailed search protocol is presented in the PRISMA flowchart, which visually represents the steps of the review process (Figure 1). All essential elements of systematic reviews outlined in the PRISMA checklist were included.

Of the 844 articles initially identified, 37 original studies, comprising 2498 patients, met the inclusion criteria for this review. Of these, 19 evaluated the effects of vitamin C supplementation on outcomes in hemodialysis patients. Six studies examined dietary vitamin C intake (Table 1), and six assessed serum or plasma vitamin C concentrations in hemodialysis patients (Table 2). Nine studies reported associations between vitamin C and markers of inflammation or oxidative stress; eight of these were interventional clinical trials (Table 3). Seven studies evaluated the influence of vitamin C on anemia, six of which assessed the effects of vitamin C supplementation (Table 4). Two studies investigated the effect of vitamin C on RLS (Table 5). We also summarized three studies that examined the impact of vitamin C supplementation on secondary hyperparathyroidism in the dialysis population (Table 6). A meta-analysis was not performed due to heterogeneity in outcome measures, substantial variability in treatment regimens and dosing, and the small sample sizes of several included studies.

## 3. Results

### 3.1. Vitamin C Intake and Dietary Deficiency

Six of the applicable studies reported data on dietary vitamin C intake among hemodialyzed patients. In four out of six studies, the intake was below the daily recommendation of 75–90 mg/day [2,8,10,11], whereas two out of six studies reported adequate dietary vitamin C intake [9,12]. Dietary habits were assessed by dietary diaries or food frequency questionnaires. One of the studies compared vitamin C intake between HD patients from China and the United Kingdom, showing substantially lower intake among Chinese patients [11].

### 3.2. Vitamin C Concentration in Serum or Plasma

Six studies evaluated serum/plasma vitamin C concentrations in the hemodialyzed population. Three of six studies included a majority of patients who reported levels below the established reference range [14,16,17]. Coveney et al. reported adequate vitamin C concentrations in patients undergoing conventional thrice-weekly (<15 h/week) hemodialysis, whereas those treated with extended-hour (>15 h/week) hemodialysis exhibited significantly lower levels [15]. A study by Kaczkan et al. showed no difference in vitamin C concentration between HD patients and healthy controls [18]. Sirover et al. demonstrated low vitamin C level in patients who did not take vitamin C supplementation; however, patients who did supplement vitamin C had adequate plasma concentration [17]. Only one out of six studies, involving 15 hemodialysis patients, reported higher vitamin C concentrations compared with healthy controls [13]. The aim of this study was to compare the effects of two dialysis membranes on oxidative stress parameters. Cuprophane dialysis was shown to induce a higher increase in oxidants and a lower compensatory increase in antioxidants when compared with polysulfone dialysis. There was no significant change in vitamin C levels between the two membranes.

### 3.3. Antioxidative and Anti-Inflammatory Properties

Studies evaluating the antioxidant potential of vitamin C have yielded mixed results. One of nine studies was observational and demonstrated an inverse association between plasma vitamin C concentrations and high-sensitivity C-reactive protein (hsCRP) [14]. Eight applicable interventional studies investigated the effects of vitamin C supplementation on inflammatory or oxidative stress markers [19,20,21,22,23,24,25,26], with six reporting a reduction in these markers [19,20,22,24,25,26]. In the study by Bogacka et al., antioxidant activity, assessed by using the Ferric Reducing Ability of Plasma (FRAP) assay, was positively correlated with vitamin C concentrations; however, data on antioxidant levels in the supplemented group was not reported [25]. Two out of eight studies showed an opposite effect. Conner et al. observed that co-administration of intravenous iron and vitamin C resulted in higher post-infusion plasma concentrations of F2-isoprostanes, IL-1, IL-10, and TNF-α compared with iron alone injections [21]. Furthermore, a small study involving 18 hemodialysis patients reported a decrease in reduced glutathione (GSH) which is an anti-oxidant marker [23].

### 3.4. Anemia and Iron Metabolism

Six out of seven available studies confirmed the efficacy of vitamin C in improving anemia-related parameters in the context of functional iron deficiency (FID). Four reports demonstrated a reduction in serum ferritin concentrations following vitamin C supplementation [28,29,32,33]. One study showed comparable improvement of FID parameters in groups receiving combined iron and vitamin C supplementation and iron alone [28]. In the study by Sultana et al., vitamin C supplementation was associated with reduced erythropoietin dose requirements [29]. The same observation was reported by Kang et al. [27]. One out of seven studies involving 39 dialysis patients showed no significant difference in the mean change in serum ferritin, serum iron, or transferrin saturation between group that received intravenous vitamin C supplementation and a control group [31].

### 3.5. Restless Legs Syndrome

We identified two clinical trials evaluating the use of vitamin C in the management of RLS in the hemodialysis population. Both Iranian trials demonstrated a reduction in RLS symptom severity following vitamin C treatment [34,35].

### 3.6. Hyperparathyroidism

Three studies assessing the effects of vitamin C supplementation on parathyroid hormone (PTH) levels were identified and are summarized in Table 6. None of these studies showed a sustained, long-term reduction in PTH. However, one study reported a significant decrease in serum phosphorus and the calcium–phosphate product in dialysis patients receiving intravenous vitamin C [37]. Meta-analysis performed by Ke et al. confirmed that vitamin C had no positive effect on mineral bone disorder in hemodialysis patients and did not influence the serum phosphorus or PTH levels [39].

There was considerable variability in the doses of vitamin C used across studies. In most of the studies vitamin C was administered orally with doses varying between 200 and 1000 mg. Intravenous supplementation was less frequent, with doses ranging from 300 to 500 mg per session.

## 4. Discussion

This review confirms the presence of vitamin C deficiency in the HD population and suggests that its supplementation might be considered in specific clinical contexts; however, the evidence for routine use is limited.

Substantial evidence supports the conclusion that vitamin C intake in HD patients is below the recommended levels. Low dietary vitamin C intake in dialysis patients may be a result of dietary restrictions imposed on this population, such as low-potassium and low-phosphate diets but also reduced appetite. However, the limitations associated with dietary recalls and food diaries should be considered, given concerns regarding their validity and reliability. Adequate dietary vitamin C intake is of significant importance, as humans, unlike other animals, are unable to synthesize this vitamin, and it plays an essential role in maintaining multiple physiological functions [40]. Another important factor that contributes to vitamin C deficiency is its loss during hemodialysis sessions [41]. Because vitamin C is completely water-soluble and has a low molecular weight of 176 Da, loss of vitamin C is substantial and is estimated at approximately 66 mg per session [42]. Loss of vitamin C does not seem to be dependent on the dialysis membrane. However, the loss appears to be smaller in patients with higher deficiency [43], which may be explained by a reduced concentration gradient between plasma and dialysate. A study by Reinmann on the effects of increased dialysis frequency on plasma vitamin C concentrations—an ancillary analysis of the randomized Frequent Hemodialysis Network (FHN) Daily Trial—reported that higher dialysis frequency (six times a week) does not lead to a further decline in vitamin C level [44], a finding somewhat unexpected. However, it is worth noticing that Coveney et al. observed significantly lower vitamin C concentrations in patients with longer extended dialysis hours in a cohort of 52 hemodialysis patients [15]. Also, laboratory assessment of vitamin C warrants further comment. Previously, vitamin C levels were most often measured using enzymatic assays and fluorometric methods, whereas today high-performance liquid chromatography (HPLC) is the method of choice due to its higher accuracy. Because different analytical techniques have been used across studies, direct comparison of vitamin C levels is challenging and should be considered as one of limitations of the present study. It should also be noted that many drugs, including non-steroidal anti-inflammatory drugs (NSAIDs) and antibiotics, may interfere with vitamin C and can lead to laboratory measurement errors. Also, instability of vitamin C in presence of light, heat and change in pH may lead to pre-analytical errors as ascorbic acid may be reversibly oxidized into dehydroascorbic acid [45].

Three of the available studies reported serum or plasma vitamin C deficiency among HD patients [14,16,17]. The study by Kaczkan et al. did not demonstrate any statistically significant differences in vitamin C levels between HD patients and the control group [18]. However, a positive correlation between plasma vitamin C and serum albumin levels was observed, suggesting that higher vitamin C concentrations may be a marker of better nutritional status, like albumin, cholesterol, and omega-3 fatty acids. Poor nutritional status, a part of the protein–energy wasting (PEW) syndrome, is a strong predictor of mortality in this population. Consequently, low total plasma vitamin C has been identified as a risk factor for cardiovascular morbidity and mortality among hemodialysis patients. In 2005, Deicher et al. conducted an observational, prospective cohort study examining the association between total plasma vitamin C levels and cardiovascular outcomes in 90 HD patients. Individuals with deficiency of vitamin C were almost fourfold higher in the context of risk for major cardiovascular events and mortality compared to patients with higher plasma levels [46]. Additionally, hemodialysis patients with comorbid conditions tend to exhibit lower vitamin C levels, and reduced plasma vitamin C concentration predicts shorter survival in this population [16]. It has also been proposed that chronic illness and state of inflammation contribute to reduction in plasma vitamin C levels [47]. Hemodialysis itself is considered a pro-inflammatory state, not only due to state of chronic uremia but also due to repeated exposure of blood to the dialysis membrane—recognized by the body as a “foreign” body—which promotes production of ROS. The antioxidant properties of vitamin C have been widely described; proposed mechanisms of the antioxidant mechanism include inhibition of nuclear factor-κB (NF-κB) expression in the kidney, which contributes to increased ROS production [48]. Vitamin C also acts as a free-radical scavenger and protects cells from oxidative stress and consecutive damage. Although vitamin C is recognized as an antioxidant, some studies have reported pro-inflammatory effects. Most studies included in this review, however, demonstrated anti-inflammatory and antioxidant properties of vitamin C. Two studies specifically assessed correlations between vitamin C levels and markers of inflammation or oxidative stress. Zhang et al. reported an inverse association between vitamin C and hsCRP, as well as a positive association with prealbumin which is a negative acute phase protein [14]. Bogacka et al. observed higher FRAP in patients with higher levels of vitamin C. FRAP is a method of measuring the antioxidant capacity in a sample [25]. Conversely, two studies suggested potential pro-inflammatory effects of vitamin C [21,23]. In one of these, an increase in pro-inflammatory markers was observed in patients receiving intravenous iron combined with vitamin C compared with those receiving iron alone [21]. However, this trial included only 13 participants, and individuals with iron overload—those who might benefit most—were excluded. It should also be noted that, unfortunately, the effect of vitamin C seems to be temporary, and withdrawal of supplementation causes the return of inflammatory markers to their original state [20]. Also, heterogeneity in dosing and administration routes makes direct comparisons between studies challenging.

Concerns have been raised about the potential accumulation of oxalate in patients with renal failure who are supplemented with vitamin C. Oxalate is a product of vitamin C metabolism and may precipitate with calcium and potentially deposit in organs leading to secondary hyperoxalosis. In a study by Ono et al. there was no beneficial effects on morbidity or mortality after two-year vitamin C supplementation, but worsening of secondary hyperoxalemia in that group was reported [49]. In subjects with normal kidney function, the excess of vitamin C is lost with urine; however, in patients with a glomerular filtration rate (GFR) below about 20 mL/min, oxalate retention increases rapidly [50]. A prospective cohort analysis by Ferraro et al. conducted on 156,735 women and 40,536 men reported that total and supplemental vitamin C intake was significantly associated with higher risk for incident kidney stones in men [51]. However, a study from 2016 by Liu et al. found that mean plasma oxalate level decreased 71% during the intradialytic period, and levels were lower than those in older studies which may indicate that earlier patients were receiving less efficient hemodialysis than nowadays [52]. Overdosing of vitamin C might also lead to temporary gastrointestinal disturbances like stomach cramps, nausea and diarrhea.

One of the major clinical challenges in the hemodialysis population is functional iron deficiency. In this population, the utilization of iron for hemoglobin synthesis is impaired due to reticuloendothelial blockade. Clinically, this condition is characterized by elevated ferritin levels with low transferrin saturation, accompanied by anemia. The role of vitamin C in mobilizing iron from tissue stores has been examined in numerous reports. Unfortunately, due to concerns regarding oxalosis, supplementation in most studies has been limited to short-term use. Nevertheless, the majority of evaluated studies demonstrate a substantial improvement in anemia management following vitamin C supplementation.

Some patients undergoing hemodialysis experience RLS, a condition with a complex and incompletely understood pathophysiology that significantly affects quality of life [53]. It is, however, well established that disturbances in iron and dopamine metabolism contribute to its development. We reviewed two small-numbered clinical trials that demonstrated efficacy of vitamin C in RLS treatment [34,35]. As previously noted, vitamin C increases the bioavailability of iron from tissue stores and possibly can alleviate RLS symptoms. In the study by Rafie and Jafari, a low dose of vitamin C was found to be as effective as pramipexole, a dopaminergic agent [35]. It is suggested that iron deficiency may reduce dopamine transporter density and diminish dopamine receptor binding [54].

In 2008, Richter et al. reported an inverse relationship between serum vitamin C levels and PTH [55]. According to the authors, vitamin C deficiency may result in end-organ resistance to PTH as it plays a role in post-receptor events including the PTH receptor in the bone. However, subsequent studies have not confirmed a beneficial effect of vitamin C on PTH concentration, and vitamin C is not recommended as a treatment for secondary hyperparathyroidism. The findings reported by Richter may instead reflect the poorer nutritional status often observed in patients with advanced secondary hyperparathyroidism.

Singer et al. showed no difference in uremic symptoms, cardiovascular stability nor in quality of life measured by Kidney Disease Quality of Life Short FORM (KDQOL-SF) after 3 months of vitamin C supplementation [56]. To our best knowledge, no long-term interventional study has demonstrated that vitamin C supplementation reduces mortality in hemodialysis patients.

## 5. Conclusions

Patients on maintenance HD are commonly deficient in serum vitamin C. However, we found no convincing evidence to support routine vitamin C supplementation for the general hemodialysis population. Studies indicate that vitamin C is a surrogate marker of malnutrition. The main limitations of our study include the heterogeneity of the reports, the poor quality of some of the included studies, low reliability of dietary questionnaires, and possible technical difficulties in measuring vitamin C serum levels.

However, we believe that the present study provides valuable insights, such as the potential beneficial role of vitamin C supplementation in restless legs syndrome and functional iron deficiency. There is a need for well-powered randomized controlled trials with long-term clinical endpoints to determine the efficacy of this intervention. While modern dialysis may mitigate oxalate accumulation, long-term safety remains insufficiently studied.

## Figures and Tables

**Table 1:** Studies on vitamin C dietary intake in hemodialysis patients.

Study	Author	Year	Country	Type of Study	Sample Size	Method	Outcome
Dietary assessment of hemodialysis patients in Tehran, Iran [8]	As’Habi A	2011	Iran	cross-sectional	291	4-day diet diary	Low vitamin C intake (35.5 ± 29 mg/day)
Dietary Intake of Vitamins in Different Options of Treatment in Chronic Kidney Disease: Is There a Deficiency? [2]	Jankowska M	2016	Poland	cross-sectional	45	24 h dietary recall	Low vitamin C intake (65.7 ± 63.7 mg/day)
Dietary intake as a predictor for all-cause mortality in hemodialysis subjects (NUGE-HD study) [9]	Balbino KP	2019	Brazil	longitudinal	85	food frequency questionnaire	Adequate vitamin C intake 185.4 mg/day (127.9–272.6)
Dietary intake of trace elements, minerals, and vitamins of patients on chronic hemodialysis [10]	Bossola M	2014	Italy	cross-sectional	128	3-day diet diary	Low vitamin C intake: 47.8 ± 50.3 mg/day
A Comparison of Dietary Intake Between Individuals Undergoing Maintenance Hemodialysis in the United Kingdom and China [11]	Song Y	2022	China	cross-sectional	83	24 h dietary recall	Low vitamin C intake (Chinese patients 39 ± 51 mg/day; UK patients 64 ± 42 mg/day)
Varying association of nutrient intakes with quality of life in patients receiving different modes of dialysis [12]	Guo Y	2024	China	cross-sectional	79	3-day diet diary	Adequate vitamin C intake 100.5 ± 76.7 mg/day

**Table 2:** Studies on vitamin C concentration in plasma/serum of hemodialysis patients.

Study	Author	Year	Country	Type of Study	Sample Size	Method	Outcome
Acute effects of hemodialysis on oxidative stress parameters in chronic uremic patients: Comparison of two dialysis membranes [13]	Ibrahim Varan H	2010	Turkey	cross-sectional	15	UV-spectrophotometric method	HD patients had higher vitamin C level than control group (1.81 ± 0.16 versus 0.59 ± 0.07)
Low levels of vitamin C in dialysis patients is associated with decreased prealbumin and increased C-reactive protein [14]	Zhang K	2011	China	cross-sectional	117	high-performance liquid chromatography (HPLC)	Vitamin C concentration 2.9 ug/mL (1.6–5.5), normal range 4–15 ug/mL
Water-soluble vitamin levels in extended hours hemodialysis [15]	Coveney N	2011	Australia	cross-sectional	52	high-performance liquid chromatography (HPLC)	Vitamin C concentrations were lower in the group of extended time on dialysis (0.30 vs. 1.14 mg/dL, *p* < 0.001), normal range: 0.48–1.5 mg/dL
Plasma vitamin C concentrations in patients on routine hemodialysis and its relationship to patients’ morbidity and mortality [16]	Dashti-Khavidaki S.	2011	Iran	prospective	91	UV-spectrophotometric method	Forty-nine patients (53.8%) had low levels of vitamin C concentration of less than 30 µmol/L
Plasma ascorbic acid concentrations in prevalent patients with end-stage renal disease on hemodialysis [17]	Sirover WD	2015	USA	prospective observational	211	high-performance liquid chromatography (HPLC)	Vitamin C concentration was 15.7 mM (8.7–66.8) in patients who did not take a supplement and 50.6 mM (25.1–88.8) in patients who did take a supplement; plasma concentration <30 mM considered abnormal
Water-Soluble Vitamins Status in Patients Undergoing Maintenance Hemodialysis [18]	Kaczkan M	2023	Poland	cross-sectional study	142	high-performance liquid chromatography (HPLC)	No difference between vitamin C concentration between dialyzed patients and control group: 1.1 (ng/μL) ± 0.79 (0.88) versus 0.89 ± 0.22 (0.93) *p* = 0.69

**Table 3:** Studies on vitamin C and oxidative stress in hemodialysis patients.

Study	Author	Year	Country	Type of Study	Sample Size	Outcome
Effect of combined vitamins C and E supplementation on oxidant/antioxidant status in hemodialysis patients [19]	Montazerifar F	2010	Iran	double-blind clinical trial	38	Increased levels of antioxidants (vitamin E, C and SOD) and GPx (*p* < 0.05) and decreased MDA concentration were induced by oral vitamin C (250 mg) and E (200IU) 2-month supplementation (three times a week)
Cross-over study of influence of oral vitamin C supplementation on inflammatory status in maintenance hemodialysis patients [20]	Zhang K	2013	China	cross-over	100	Decreased level of hs-CRP and an increased level of prealbumin were induced by oral vitamin C (200 mg a day) supplementation for 3 months in both groups
Effect of intravenous vitamin C on cytokine activation and oxidative stress in end-stage renal disease patients receiving intravenous iron sucrose [21]	Conner TA	2012	USA	cross-over	13	Increased plasma concentrations of F2-isoprostanes, IL-1, IL-10, and TNF-α after intravenous infusion of iron sucrose with vitamin C (300 mg) compared to iron sucrose (concentrations were measured post-infusion)
Low levels of vitamin C in dialysis patients is associated with decreased prealbumin and increased C-reactive protein [14]	Zhang K	2011	China	cross-sectional	117	Plasma vitamin C level was inversely associated with hsCRP concentration (Spearman r = −0.201, *p* = 0.001) and positively associated with prealbumin (Spearman r = 0.268, *p* < 0.001), albumin levels (Spearman r = 0.161, *p* = 0.007).
Effect of Vitamin C Supplementation on C-reactive Protein Levels in Patients Undergoing Hemodialysis: A Randomized, Double Blind, Placebo-Controlled Study [22]	Biniaz V	2014	Iran	randomized, clinical trial	151	Decreased level of CRP was induced by intravenous vitamin C supplementation (250 mg three times a week for 2 months)
Vitamin C decreases reduced glutathione in chronic hemodialysis patients: a pilot, randomized, double-blind trial [23]	Martins ML	2021	Brazil	randomized, clinical trial	18	Decreased GSH levels induced by oral vitamin C supplementation (250 mg)
Evaluation of the combination effect of rutin and vitamin C supplementation on the oxidative stress and inflammation in hemodialysis patients [24]	Omar S	2022	Egypt	randomized, clinical trial	105	Increased level of GPx induced by oral vitamin C supplementation (1000 mg)
Effect of Diet and Supplementation on Serum Vitamin C Concentration and Antioxidant Activity in Dialysis Patients [25]	Bogacka A	2023	Poland	clinical trial	68	A statistically significant positive correlation was observed between the vitamin C concentration and FRAP (correlation coefficient r = 0.37)
Whey Protein, Vitamins C and E Decrease Interleukin-10 in Chronic Hemodialysis Patients: A Pioneer, Randomized, Double-Blind Pilot Trial [26]	da Silva AT	2024	Brazil	randomized, clinical trial	23	Decreased level of IL-10 induced by oral vitamin C supplementation (250 mg three times a week for 2 months), and there was no statistically significant difference in other inflammatory markers

IL, Interleukin; FRAP, The Ferric Reducing Ability of Plasma; GSH, Reduced glutathione; GPx, Glutathione peroxidase; hs-CRP, High-sensitivity C-reactive protein; MDA, Malondialdehyde; SOD, Superoxide dismutase.

**Table 4:** Studies on vitamin C supplementation/concentration impact on anemia in hemodialysis patients.

Study	Author	Year	Country	Type of Study	Clinical Scenario	Sample Size
The effect of intravenous ascorbic acid in hemodialysis patients with normoferritinemic anemia [27]	Kang D	2012	Gwangju Korea	randomized, clinical trial	Anemia improvement and EPO requirement decline after intravenous Vitamin C supplementation (500 mg for 3 months)	58
Comparative study of intravenous iron versus intravenous ascorbic acid for treatment of functional iron deficiency in patients under hemodialysis: A randomized clinical trial [28]	Sedighi O	2013	Iran	randomized, clinical trial	FID improvement after intravenous iron (100 mg/twice a week for 5 weeks) and intravenous vitamin C (300 mg/twice a week for 5 weeks) supplementation is comparable	40
Oral Vitamin C Supplementation Reduces Erythropoietin Requirement in Hemodialysis Patients with Functional Iron Deficiency [29]	Sultana T	2015	USA	clinical trial	Oral vitamin C supplementation (250 mg/day for 3 months) reduced EPO dose requirements in hemodialysis patients with FID	22
Plasma vitamin C levels in ESRD patients and occurrence of hypochromic erythrocytes [30]	Seibert E	2017	USA	cross-sectional study	High plasma levels of vitamin C were negatively associated with hypochromic RBC	149
Impact of vitamin C supplementation on serum ferritin level in hemodialysis patients [31]	Bashardoust B	2018	Iran	randomized, clinical trial	There was no significant difference in the mean change in serum ferritin between group that received intravenous vitamin C supplementation (500 mg twice a week for 2 months) and a control group	39
The Effect of Intravenous vitamin C on Ferritin Levels in Patients Hemodialysis Patients, A Clinical Trial [32]	Hajian S	2022	Iran	randomized, clinical trial	Decreased ferritin level after intravenous supplementation with vitamin C (500 mg/3 days a week during 3 months)	32
Role of Intravenous Ascorbic Acid in the Management of Anemia in Hemodialysis Patients [33]	El Shinnawy H	2023	Egypt	randomized, clinical trial	Increased hemoglobin level and decreased ferritin level in a group receiving intravenous sucrose iron (100 mg once a week) and vitamin C (500 mg three times a weeks) for 6 months supplementation compared to a group with discontinuation of iron therapy	50

FID, functional iron deficiency; EPO, erythropoietin.

**Table 5:** Studies on vitamin C supplementation and RLS.

Study	Author	Year	Country	Type of Study	Sample Size	Outcome
Efficacy of vitamins C, E, and their combination for treatment of restless legs syndrome in hemodialysis patients: a randomized, double-blind, placebo-controlled trial [34]	Sagheb MM	2012	Iran	a randomized, double-blind, placebo-controlled trial	60	Vitamin C (200 mg/day) and vitamin E (400 mg/day) and their combination for 2 months are safe and effective treatments for reducing the severity of RLS in hemodialysis patients
A Comparative study on the effects of vitamin C and Pramipexole on restless legs syndrome treatment in hemodialysis patients: A randomized, double blind, placebo-controlled trial [35]	Rafie S and Jafari M	2016	Iran	double-blind, placebo-controlled trial	45	Vitamin C (250 mg/day orally) was as effective as pramipexole (0.18 mg/day orally) in RLS treatment

**Table 6:** Studies on vitamin C supplementation and parathyroid hormone level.

Study	Author	Year	Country	Type of Study	Sample Size	Clinical Scenario
Effect of vitamin C on parathyroid hormone in hemodialysis patients with mild to moderate secondary hyperparathyroidism [36]	Sanadgol H	2011	Iran	clinical trial	21	There was a decrease in PTH level initially, but there was no difference in PTH level after 3-month intravenous supplementation (250 mg/3 times a week)
The effect of intravenous vitamin C on the phosphorus level reduction in hemodialysis patients: A double blind randomized clinical trial [37]	Baradari AG	2012	Iran	a double-blind randomized clinical trial	60	There was a significant decrease in phosphorus (*p* = 0.01), CRP level (*p* = 0.01) and Ca × P product (*p* = 0.03) in a group treated with intravenous vitamin C (500 mg/3 times a week for 2 months)
The effect of vitamin C on parathyroid hormone in patients on hemodialysis with secondary hyperparathyroidism: A double blind, placebo-controlled study [38]	Biniaz V	2013	Iran	A double-blind, placebo-controlled study	82	There was no difference in decrease in PTH level after 2-month intravenous supplementation (200 mg/3 times a week) and control group

PTH, parathyroid hormone; Ca, calcium; P, phosphorus.

## Data Availability

No new data were created or analyzed in this study. Data sharing is not applicable to this article.

## Abbreviations

The following abbreviations are used in this manuscript:

CKD	Chronic kidney disease
GSH	Reduced glutathione
FID	Functional iron deficiency
FRAP	Ferric Reducing Ability of Plasma
GFR	Glomerular filtration rate
HD	Hemodialysis
hsCRP	High-sensitivity C-reactive protein
PTH	Parathyroid hormone
RLS	Restless legs syndrome
ROS	Reactive oxygen species
